# Monthly Record of Deaths in the City of Buffalo

**Published:** 1858-04

**Authors:** H. D. Garvin

**Affiliations:** Health Physician


ART. VI.— *Report of Mortality in Buffalo for the month of Feb.*, 1858 By H. D. Garvin, M. D., Health Physician.

| DISEASES. | No. | Males. | Females. | No Sex given. |
|---|---|---|---|---|
| Accident, | 2 | 1 | 1 | |
| Bronchitis, | 2 | 1 | 1 | |
| Bright's Disease, | 1 | | 1 | |
| Cirrhosis of Liver, | 1 | | 1 | |
| Congestion of Lungs, | 1 | | 1 | |
| " " Brain, | 1 | 1 | | |
| Convulsions, | 17 | 11 | 6 | |
| Croup, | 3 | 2 | 1 | |
| Debility, | 4 | 2 | 2 | |
| Disease of Brain, | 1 | 1 | | |
| Diarrhœa, | 1 | | 1 | |
| Dropsy, | 6 | 2 | 4 | |

| DISEASES. | No. | Males. | Females. | No Sex given. |
|---|---|---|---|---|
| Delirium Tremens, | 2 | 2 | | |
| Dysentery, | 2 | 1 | 1 | |
| Enteritis, Acute, | 2 | 2 | | |
| Epilepsy, | 1 | | 1 | |
| Fever, Typhoid, | 1 | | 1 | |
| " Typhus, | 1 | 1 | | |
| " Scarlet, | 2 | 2 | | |
| Gastritis Chronic, | 1 | | 1 | |
| Gangrena Senilis, | 1 | | 1 | |
| Heart Disease, | 2 | 1 | 1 | |
| Hereditary Syphilis, | 1 | 1 | | |
| Hydrocephalus, | 8 | 5 | 3 | |
| Laryngitis, | 2 | 2 | | |
| Marasmus, | 5 | 2 | 3 | |
| " Senile, | 1 | | 1 | |
| Meningitis, | 1 | 1 | | |
| Medullary Sarcoma. | 1 | | 1 | |
| Obstruction of Bowels, | 1 | | 1 | |
| Old Age, | 4 | 1 | 3 | |
| Phthisis Pulmonalis, | 15 | 8 | 7 | |
| Pneumonia, | 2 | 1 | 1 | |
| Premature Ossification and Closure of Bones of Head, | 1 | 1 | | |
| Pyæmia, | 1 | 1 | | |
| Still-born, | 4 | 3 | 1 | |
| Teething, | 2 | | 2 | |
| Tabes Mesenterica, | 1 | | 1 | |
| Whooping Cough, | 2 | 1 | 1 | |
| No Cause of Death given, | 9 | | | |
| Total, | 116 | 56 | 51 | |

SEXES.

| | |
|---|---|
| Males, | 56 |
| Females, | 51 |
| Sex not given, | 9 |
| Total, | 116 |

AGES.

| | | | |
|---|---|---|---|
| Still-born, | 4 | Between 20 years and 30 years, | 8 |
| 1 day, | 4 | " 30 " " 40 " | 6 |
| 1 day and 30 days, | 7 | " 40 " " 50 " | 8 |
| Between 1 month and 6 months, | 16 | " 50 " " 60 " | 2 |
| " 6 months and 12 " | 13 | " 60 " " 70 " | 5 |
| " 1 year " 3 years, | 16 | " 70 " " 80 " | 5 |
| " 3 " " 5 " | 8 | " 80 " " 90 " | 1 |
| " 5 " " 10 " | 4 | " 90 " " 100 " | 0 |
| " 10 " " 20 " | 7 | " 100 " | 0 |
| | 77 | | 35 |
| Ages not given, | 3 | | 112 |
| Total, | 144 | | |

NATIVITIES.

| | | | |
|---|---|---|---|
| American, (white,) | 69 | Prussian, | 0 |
| " (colored,) | 8 | Swiss, | 1 |
| German, | 13 | French, | 3 |
| Irish, | 12 | Scotch, | 1 |
| English, | 2 | Bohemia. | 1 |
| Canadian, | 0 | Country not given, | 5 |
| Total, | | | 11 |

